# Coincidence of photic zone euxinia and impoverishment of arthropods in the aftermath of the Frasnian-Famennian biotic crisis

**DOI:** 10.1038/s41598-019-52784-4

**Published:** 2019-11-18

**Authors:** Krzysztof Broda, Leszek Marynowski, Michał Rakociński, Michał Zatoń

**Affiliations:** 1 Department of Palaeontology and Stratigraphy, University of Silesia in Katowice, Faculty of Earth Sciences, Będzińska 60, 41-205 Sosnowiec, Poland; 2 Department of Geochemistry, Mineralogy and Petrography, University of Silesia in Katowice, Faculty of Earth Sciences, Będzińska 60, 41-205 Sosnowiec, Poland

**Keywords:** Ecology, Biogeochemistry, Environmental sciences

## Abstract

The lowermost Famennian deposits of the Kowala quarry (Holy Cross Mountains, Poland) are becoming famous for their rich fossil content such as their abundant phosphatized arthropod remains (mostly thylacocephalans). Here, for the first time, palaeontological and geochemical data were integrated to document abundance and diversity patterns in the context of palaeoenvironmental changes. During deposition, the generally oxic to suboxic conditions were interrupted at least twice by the onset of photic zone euxinia (PZE). Previously, PZE was considered as essential in preserving phosphatised fossils from, e.g., the famous Gogo Formation, Australia. Here, we show, however, that during PZE, the abundance of arthropods drastically dropped. The phosphorous content during PZE was also very low in comparison to that from oxic-suboxic intervals where arthropods are the most abundant. As phosphorous is essential for phosphatisation but also tends to flux off the sediment during bottom water anoxia, we propose that the PZE in such a case does not promote the fossilisation of the arthropods but instead leads to their impoverishment and non-preservation. Thus, the PZE conditions with anoxic bottom waters cannot be presumed as universal for exceptional fossil preservation by phosphatisation, and caution must be paid when interpreting the fossil abundance on the background of redox conditions.

## Introduction

Euxinic conditions in aquatic environments are defined as the presence of H_2_S and absence of oxygen^1^. If such conditions occur at the chemocline in the water column, where light is available, they are defined as photic zone euxinia (PZE). In these oxygen-free and hydrogen sulphide-rich environments, some bacteria (e.g., purple sulphur bacteria or green sulphur bacteria) can thrive and photosynthesise, thereby playing a crucial role in the sulphur cycle in stratified basins^2^. In ancient sedimentary rocks, anoxygenic photosynthesis and thus the presence of PZE are best evidenced by the presence of specific ‘molecular fossils’ (biomarkers), which are generated by anaerobic and photosynthetic bacteria^3–5^. PZE was an important environmental factor during the major extinction events in the Earth’s history, especially during oceanic anoxic events such as the end-Ordovician^6^, latest Devonian^5^ and end-Permian^7^. The PZE has also been proposed as a factor leading to mass mortality events of fish preserved in the Cretaceous Santana Formation^8^. Recently, researchers have emphasised that PZE played a crucial role in the exceptional preservation of phosphatic fossils in the famous Upper Devonian conservation deposits of the Gogo Formation in Australia^2^.

In this paper, we document the recurrent PZE conditions during sedimentation of the fossiliferous lower Famennian (Upper Devonian) deposits at the Kowala quarry (Holy Cross Mountains, Poland). Because of the great abundance and excellent preservation of phosphatic fossils of thylacocephalan, phyllocarid and angustidontid crustaceans^9–12^, articulated coelacanth fish^13^, conulariids^14^, coprolites^15^, as well as carbonaceous non-biomineralised algae^16^, these deposits were coined as the Kowala Lagerstätte^9^. Interestingly, these fossiliferous deposits originated during the earliest Famennian, so in the aftermath of the famous Frasnian-Famennian (F-F) biotic crisis, during which many marine groups suffered extinction and, especially, reef ecosystems collapsed^17,18^. Although intense volcanism has been considered as the most probable ultimate cause of the F-F event (e.g.^19,20^), the hypotheses concerning the exact proximate kill mechanism of the crisis differ^21^. However, the common association of black “Kellwasser” facies near the F/F boundary^22^ supports the event might have been related to the transgression-related anoxia^23,24^.

Combining palaeontology and geochemistry, we show that after the F-F biotic crisis, the PZE still occurred intermittently during the early Famennian in the Late Devonian shelf basin of the southern margin of the Laurussia continent (present day Holy Cross Mountains, Poland). We also show that PZE did not lead to mass mortality events of arthropods, which abundantly inhabited the basin and, importantly, have not promoted their fossilisation as was considered previously. Our study shows that euxinic conditions should not be used as universal explanation for exceptional fossil preservation. Instead, a strong caution must be paid when interpreting the taphonomy of phosphatised fossils and fluctuations of their abundance in the rock record.

## Geological Background

**Figure 1:**
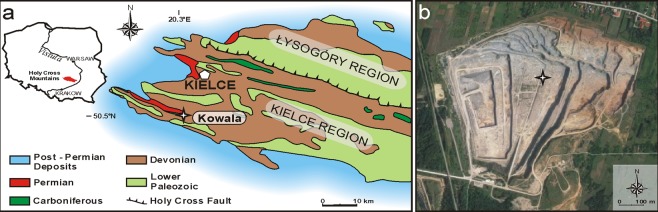
Locality of the studied section. (**a**) Location of the Kowala quarry on the background of the geology of the Holy Cross Mountains; (**b**) The Kowala quarry with an exact position of the investigated section indicated (Google Maps, 2019).

The active Kowala quarry is located in the southern limb of the Gałęzice-Bolechowice syncline in the southern part of the Kielce region of the Holy Cross Mountains (Fig. 1). During the Devonian, the Holy Cross Mountains area was part of a carbonate shelf that extended along the southern margin of the continent of Laurussia near the equator^25–29^. The Famennian sediments of the Kowala quarry were deposited in the intrashelf Chęciny-Zbrza basin^28,30,31^. The lower Famennian deposits investigated in this paper are 21 m thick and crop out in a trench located in the north-central part of the quarry (N50°47′43,476′′, E20°33′53,568′′, Fig. 1). The section comprises monotonous deposits of thin-bedded, dark, carbonaceous shales and thin-bedded, grey, micritic limestones. The investigated interval is confined to lithologic unit H-4 of Racki & Szulczewski^32^, which stratigraphically encompasses the Late *triangularis* through Early *marginifera* conodont zones^32^. Earlier, the lower Famennian interval investigated in this paper was assumed as being confined to the *crepida* conodont Zone^9,15^, on the basis of lithological similarities and its position relative to the neighbouring trench section investigated by Marynowski *et al*.^33^. However, the new conodont dating showed that our section is slightly older and represents the *Palmatolepis minuta minuta* Zone (according to the latest conodont zonation of Spalletta *et al*.^34^), which corresponds to the previously established Late *triangularis* conodont Zone of Ziegler & Sandberg^35^. The conodont assemblage includes: *Palmatolepis protorhomboidea*, *Pa*. *delicatula delicatula*, *Pa*. *superlobata*, *Polygnathus brevilaminus*, *Po*. *procerus*, *Icriodus alternatus alternatus*, and *Ic*. *deformatus deformatus*.

The depositional environment is generally interpreted as deep shelf, below storm wave-base, but at least episodically within the limits of the photic zone^33,36–38^.

## Material and Methods

### Collection of fossils

We collected 2029 specimens in total directly from the investigated section. At least 32 specimens were collected from each of the 34 investigated shale or marly shale beds, regardless of the preservation state or systematic position of the specimens. The specimens were not retrieved from limestones as they are much harder to work with because they are very likely to be damaged while splitting the rock.

For the purposes of this study, fossilised regurgitates, coprolites, and trace fossils were excluded, as they represent the signs of animal activity, and not the animal itself. In some cases, the total abundance of some body fossils within each bed was difficult to establish because their skeletons tend to disarticulate post-mortem (crinoids, coelacanth fish) or were extremely rare (angustidontid arthropods); these fossils were marked on diagrams but not evaluated quantitatively. Finally, we included 1840 of the 2029 specimens collected *in situ* in our quantitative palaeoecological analyses.

Since during the fieldwork, obtaining the same number of specimens for each bed was not always achievable and some of the collected specimens were excluded from the study, the general number of each fossil type in each bed was converted to a percentage contribution. Such a calculation allowed for comparisons with all the needed proportions being saved, despite the differences in total number of specimens between the studied beds.

### Total organic carbon (TOC) and total sulphur (TS) content

For total carbon (TC), total inorganic carbon (TIC) and total sulphur (TS) measurements, an ELTRA CS-500 IR-analyser was used (at Faculty of Earth Sciences, University of Silesia in Katowice, Poland). TOC was calculated as the difference between TC and TIC. ELTRA standards were applied for the calibration. Values of better than ±2% for TC and ±3% for TIC were observed for analytical precision and accuracy (for more details see^5^).

### Extraction, separation and derivatisation

An extraction of ground samples (<100 mesh) was performed using an accelerated Dionex ASE 350 solvent extractor by a mixture of dichloromethane (DCM)/methanol (1:1 v:v). For the extracts’ separation, micro-column (on Pasteur pipettes) chromatography was used. Silica gel was activated at 120 °C for 24 h, then cooled and poured into micro-columns. Extracts were separated into three fractions: aliphatic, aromatic, and polar, using: *n*-pentane, *n*-pentane and DCM (7:3 v:v), and DCM and methanol (1:1 v:v), respectively (for more details see^39^). For maleimide analysis, the polar fraction was again separated in micro-columns, using a DCM/acetone mixture (8:2 v:v). Then, a DCM/acetone fraction was derivatised by MTBSTFA (N-tert-butyldimethylsilyl-N-methyltrifluoroacetamide). Samples were derivatised with MTBSTFA dissolved in super-dehydrated DCM, heated at 50 °C for 1 h, and analysed right after derivatisation. After derivatisation, tert-butyl-dimethylsilyl derivatives of maleimides were obtained (see also^40^ and^41^).

### Gas chromatography coupled with mass spectrometry (GC-MS)

GC-MS analyses were carried out with an Agilent Technologies 7890 A gas chromatograph and Agilent 5975 C Network mass spectrometer with a Triple-Axis Detector (MSD). Separation was obtained on a fused silica capillary column (J&W HP5-MS, 60 m x 0.25 mm i.d., 0.25 µm film thickness) coated with a chemically bonded phase (5% phenyl, 95% methylsiloxane). The GC oven temperature was programmed from 45 °C (1 min) to 100 °C at 20 °C/min, and then to 300 °C at 3 °C/min (hold 80 min), with a solvent delay of 10 min. Helium was used as a carrier gas at a constant flow of 2.6 ml/min. Analyses were performed at the Faculty of Earth Sciences,, University of Silesia in Katowice, Poland. For more details see^41^.

### Inorganic geochemistry

The 34 pulp samples were analysed at Bureau Veritas Acme Labs Canada Ltd. Major, minor, and trace elements were analysed using inductively coupled plasma optical emission spectrometry (ICP-OES) and inductively coupled plasma mass spectrometry (ICP-MS) (details described in^42^). The precision and accuracy of the results were better than ±0.05% (mostly ± 0.01%) for the major elements and generally better than ±1 ppm for the trace elements.

## Results

### Abundance and composition of fossil assemblages

**Figure 2:**
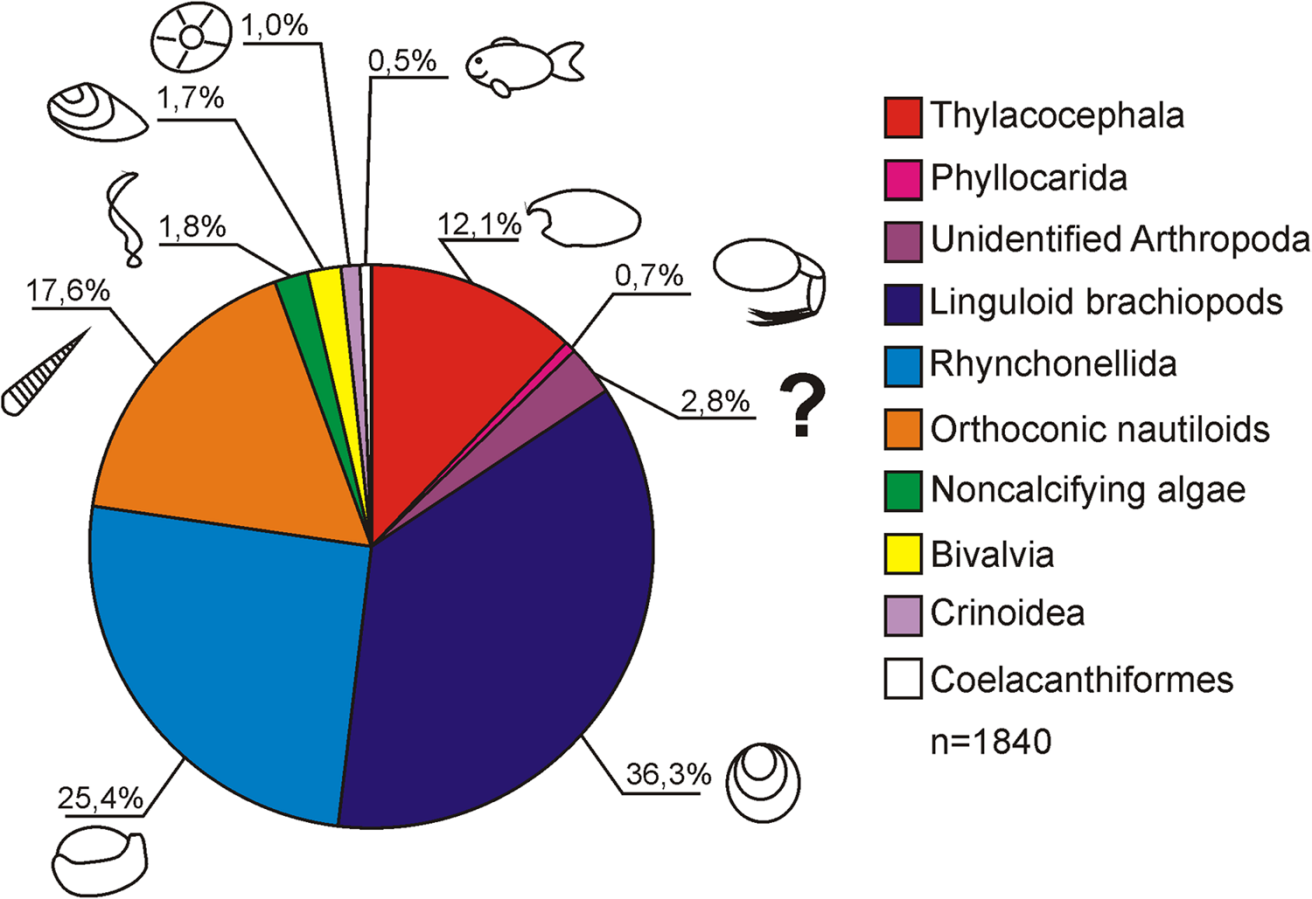
Percentage contribution of the investigated lower Famennian body fossils from the Kowala quarry. Only specimens found *in situ* are included. Note that brachiopods (linguloid + rhynchonellid) dominate the assemblage.

**Figure 3:**
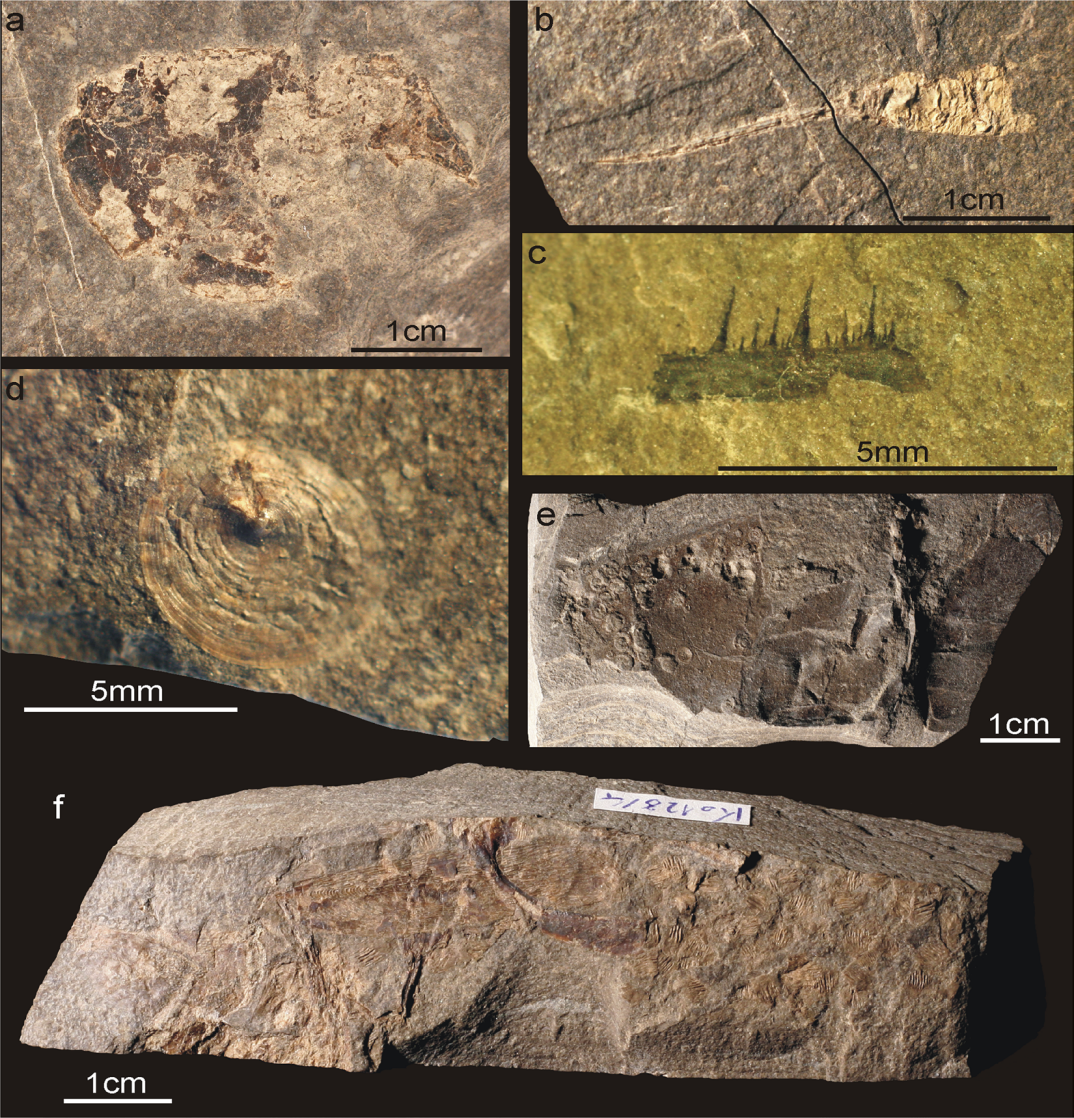
Examples of fossils from the investigated lower Famennian interval. Arthropods: (**a**) Carapace of thylacocephalan *Concavicaris* sp. aff. *bradleyi* (GIUS 4-3622/Th154a), (**b**) Pleon and telson of phyllocarid *Echinocaris bisulcata* Broda et al. 2019 (GIUS 4-3622/120-6), (**c**) Fragment of maxillipede of *Angustidontus* sp. (GIUS 4-3622/Kor110). Other fossils: (**d**) Shell of inarticulate brachiopod *Orbiculoidea* sp. (GIUS 4-3622/Kow190B/3 C), (**e**) Carbonaceous imprint of non-calcifying alga covered by numerous microconchid tubeworms (GIUS 4-3622/Kow190A/12), (**f**) Clustered bones and scales of a coelacanth fish (GIUS 4-3654/21).

**Figure 4:**
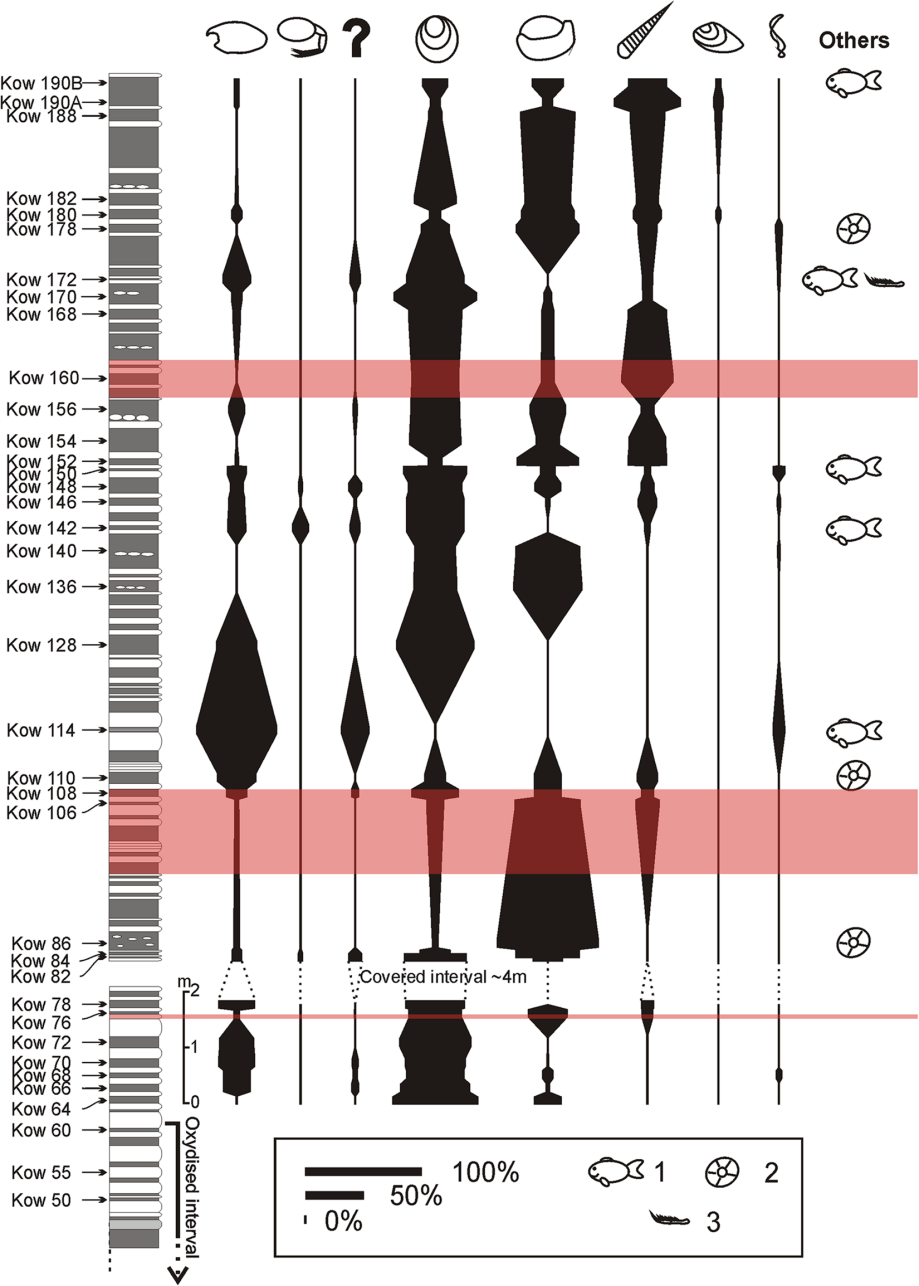
Spindle diagrams showing the relative abundances of the main fossil groups from each investigated bed. On the same right side, the occurrences of Coelacanthiformes (1), Crinoidea (2), and Angustidontidae (3) are indicated. Euxinic levels are shaded. See Fig. 2 for an explanation of the symbols.

In the studied assemblages (Figs 2 and 3), the most common fossils consisted of phosphatic shells of linguloid (*Orbiculoidea* sp.; 36.3%) and calcitic shells of rhynchonellid (25.4%) brachiopods. Interestingly, when *Orbiculoidea* brachiopods dominate, the relative abundance of rhynchonellids lowers and *vice versa* (Fig. 4). The next most abundant assemblages are orthoconic nautiloids (17.6%) preserved in the form of carbonaceous imprints of their periostracum or poorly preserved internal moulds. In general, they are more abundant in younger beds (Fig. 4). The first small peak in their percentage contribution occurs in beds Kow75 and Kow77 (12.5% and 13.33% respectively). The next peak in relative abundance occurs in beds Kow106-Kow110 (max 20.69%). From bed Kow136 on, nautiloid remains are present in every investigated bed. From that point on, their overall abundance gradually rises (with some minor fluctuations). In the lower part of bed Kow190 (Kow190A), their percentage contribution reaches the highest value of 63.46%. This and Kow160 are the only beds where nautiloids are the most abundant component of the assemblage.

Arthropods preserved as phosphatised carapaces are the third most abundant group. Concavicarid thylacocephalans (12.1%) and unidentified (due to fragmentation and/or preservation state) arthropod remains (most probably Concavicarididae as well; 2.8%) are the most common arthropods. The rarest arthropods of these units are phyllocarids with only 13 fossils (0.7%) found *in situ* and one single Angustidontidae (maxillipedes).

Non-calcifying algae (1.8%), preserved as carbonaceous compressions, the bivalve *Guerichia* sp. (1.7%), crinoid ossicles (1.0%), and coelacanth fish bone clusters or isolated elements (0.5%), are all very rare (Figs 2 and 4). Thus, they have not been included in quantitative analyses.

### Changes in arthropod abundance within the assemblages

In general, the percentage contribution of arthropod fossils decreases towards the younger beds (Fig. 4). The most abundant arthropods are representatives of the Concavicarididae (Thylacocephala), whose abundance is characterised by several rapid changes with maximally 8%. Unidentified arthropod remains (the majority may represent Thylacocephala, as well) follow the same pattern as Thylacocephala fossils, with their maxima in the same beds (Fig. 4 and Table 3). In the case of Phyllocarida, their relative abundance rises only in four intervals. In the intervals between these beds, no phyllocarid remains were found (Fig. 4 and Table 1).

**Table 1:** Relative abundances of arthropod groups (Thylacocephala, unidentified arthropod remains and Phyllocarida) in each of the investigated beds.

Bed	Thylacocephala	Unid. Arthropoda	Phyllocarida	Total
Kow 190B	6.02	2.41	0	8.43
Kow 190A	6.73	0.96	0	7.69
Kow 188	0	1.63	0	1.63
Kow 182	4.35	0	0	4.35
Kow 180	12.12	0	0	12.12
Kow 178	1.09	0	1.09	2.18
Kow 172	24.44	11.11	0	35.55
Kow 170	11.86	3.39	0	15.25
Kow 168	8.62	0	0	8.62
Kow 160	1.61	0	0	1.61
Kow 156	13.21	5.66	0	18.87
Kow 154	1.89	1.89	0	3.78
Kow 152	4.08	0	0	4.08
Kow 150	16	0	2	18
Kow 148	9.09	9.09	5.46	23.64
Kow 146	16.49	4.12	2.06	22.67
Kow 142	16.67	10	13.33	40
Kow 140	0	0	0	0
Kow 136	0	0	1	1
Kow 128	33.33	0	0	33.33
Kow 114	68.18	18.18	0	86.36
Kow 110	33.33	2.56	0	35.89
Kow 108	16.67	6.67	0	23.34
Kow 106	3.45	0	0	3.45
Kow 86	5	0	0	5
Kow 84	8.82	5.88	0	14.7
Kow 82	8	12	4	24
Kow 78	33.33	0	0	33.33
Kow 76	6.25	2.08	0	8.33
Kow 72	31.94	1.39	0	33.33
Kow 70	33.33	7.69	0	41.02
Kow 68	24.19	4.84	0	29.03
Kow 66	24.53	7.55	0	32.08
Kow 64	0	0	0	0

The grey colour indicates the maximum percentage contribution of arthropods in comparison to the background values.

**Figure 5:**
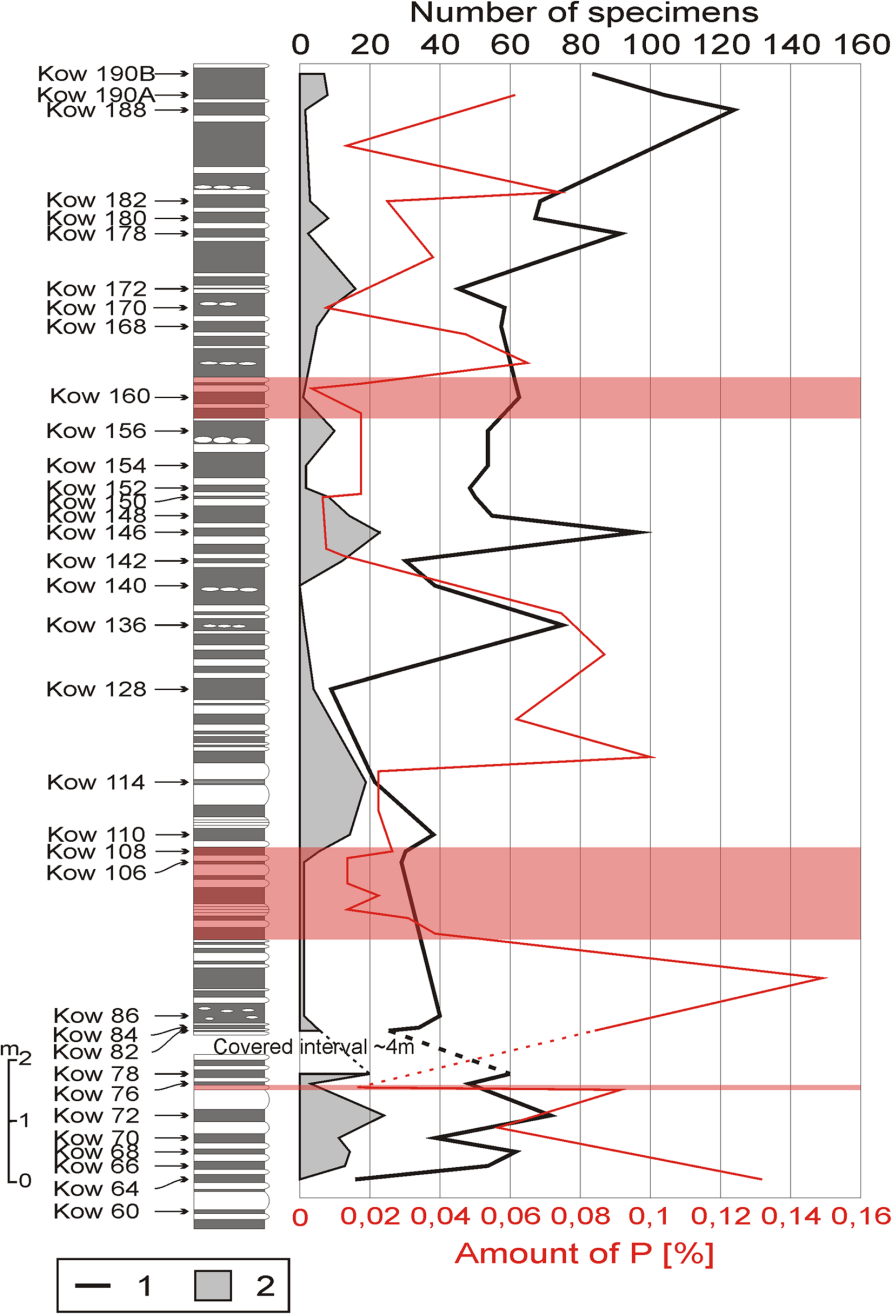
Changes in the abundance of body fossils in the investigated section. Abbreviations: 1, total number of body fossils; 2, number of arthropod fossils (Thylacocephala + Phyllocarida + unidentified arthropod remains). The euxinic levels are shaded. The amount of phosphorous in particular samples is marked with a red line.

These changes are also reflected in the total number of the collected arthropod specimens (Fig. 5). The number of specimens found *in situ* ranges from 0 (beds Kow 64, Kow 140) to 24 (bed Kow 72) and, in general, it gradually lowers towards the younger beds with tendency to differ between neighboring beds (Fig. 5). However, in some cases, when total number of acquired specimens lowers in the same beds, the percentage contribution of arthropods rises (Fig. 5). Specimen numbers per sample range between 9 (bed Kow 128) and 123 (bed Kow 188). Generally, the fossil abundance rises towards the younger beds. However, three peaks of increased abundance (compared to the overall trend) were found: beds Kow 66–77, Kow 128–152 and Kow 172–190 b. In the lower part of the section (Kow 64–77), the changes in arthropod and total specimen numbers are simultaneous and similar in their amplitudes (the relative abundance does not change much). This applies also to the first rise in fossil abundance (Kow 106–114). The first part of the second major peak of abundance (Kow 128–142) is, however, characterised by a drop in arthropod abundance. In bed Kow 140, we found no arthropod remains. Then, in the second part of this peak (Kow 142–152), their abundance rises following the general trend, but with a much lower amplitude. In the last major peak, the number of arthropod specimens again drops gradually, with some minor peaks in beds Kow 172 (beginning of the total peak), Kow 180 and Kow 190; these changes, however, follow the opposite trend, i.e. the arthropod number rises while at decreasing sample size.

### Organic geochemical data

**Figure 6:**
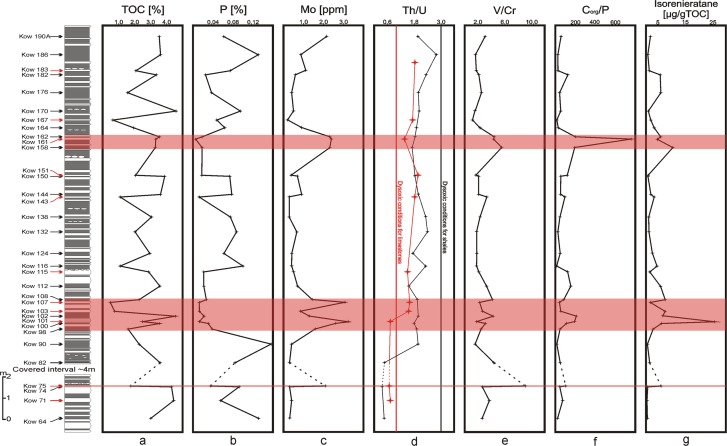
Composite plot of the lower Famennian section showing organic carbon, as well as inorganic and biomarker proxy data. (**a**) Total organic carbon content — TOC [%], (**b**) Total phosphorus content [%], (**c**) Total molybdenum content [ppm], (**d**) The thorium to uranium ratio, (**e**) The vanadium to chromium ratio, (**f**) Total organic carbon to total phosphorus ratio, (**g**) Isorenieratane concentration (μg/g TOC). Red arrowsindicate limestones while black arrows indicate mudstones.

The results are presented in Table 2 and Fig. 6. Total organic carbon values are in a rather narrow range from 1.2% wt. to 4.6% wt. in our samples. In general, samples that are rich in carbonate are slightly depleted in organic carbon, but differences in TOC content between marls and shales are minor. The section is characterised by a low total sulphur concentration between 0% and 0.5% wt., but in most of the samples, it is below 0.1% wt.

**Table 2:** Aryl isoprenoids, isorenieratane and palaeorenieratane concentrations, steranes and hopanes ratios and the 2-methyl-3-iso-butyl- to 2-methyl-3-ethyl-maleimide ratio.

Sample	Aryl isoprenoids sum µg/gTOC	2,3,6 diaryl isopren. µg/gTOC	Isorenieratane µg/g TOC	Ster/17α-hop	G/H	Me,i-Bu/Me,Et x1000
K190	11.24	2.15	1.83	0.75	0.04	1.68
K186	3.08	0.61	0.68	0.69	0.02	3.45
K183L	3.93	0.97	1.05	0.63	0.04	2.25
K182	3.26	6.08	5.21	0.64	0.03	2.42
K176	18.13	5.90	5.39	0.61	0.05	2.63
K170	2.74	1.09	1.14	0.62	0.03	2.76
K167L	2.94	2.57	2.09	0.61	0.04	0.00
K164	3.77	2.53	2.90	0.75	0.04	0.00
K162	9.93	5.36	5.69	0.73	0.07	2.96
K161L	6.64	4.36	4.85	0.67	0.07	1.03
K158	3.47	9.28	10.01	0.78	0.06	1.98
K151L	5.70	1.08	1.27	0.61	0.03	2.11
K150	4.87	1.06	1.13	0.76	0.03	2.46
K144	3.08	1.71	1.81	0.60	0.02	3.25
K143L	7.81	2.43	2.97	0.57	0.03	1.15
K138	4.36	1.17	1.32	0.72	0.03	2.38
K132	6.33	1.15	1.32	0.52	0.01	1.83
K124	8.40	2.24	2.32	0.63	0.03	3.01
K116	14.11	4.26	4.72	0.68	0.03	2.44
K115L	7.43	1.16	1.36	0.59	0.04	1.60
K112	8.10	3.89	5.22	0.59	0.04	2.31
K108	5.84	4.47	7.13	0.73	0.04	4.85
K107L	0.95	1.37	1.80	0.56	0.06	0.00
K103L	2.20	5.07	7.13	0.59	0.06	0.00
K102	10.75	4.15	6.46	0.61	0.05	2.53
K101L	30.00	10.09	26.02	0.72	0.09	0.90
K100	7.95	3.12	5.50	0.69	0.08	3.37
K98	5.41	3.08	2.55	0.60	0.08	3.85
K90	7.58	0.49	0.36	0.56	0.04	3.90
K82	3.14	1.07	1.17	0.54	0.06	2.80
K75L	12.73	7.51	6.29	0.49	0.13	1.05
K74	3.42	0.49	0.55	0.51	0.06	2.98
K71L	3.24	0.35	0.33	0.49	0.07	1.97
K64	3.14	0.42	0.44	0.48	0.07	3.34

Ster/17α-hop = steranes consist of the C_27_, C_28_, C_29_ ααα(20 S + 20 R), αββ(20 S + 20 R) and diasteranes, 17α-hopanes consist of the C_27_ to C_35_ pseudohomologues (with 22 S and 22 R epimers). G/H = Gammacerane/C_30_ 17α-hopane ratio.

Steranes to hopanes ratio values are in the range of 0.5 to 0.8 (Table 2) which is similar to other parts of the Kowala section, including *Annulata* and Dasberg^42,43^, and lower than values noted for the Hangenberg secion^5^. These data imply that both algae and bacteria were important organisms contributing to the kerogen formation. Gammacerane, an indicator of water column stratification, was found in the all samples with relative concentration also compatible to the other sections from the Kowala quarry^42,43^. Despite the small differences in the TOC content, important changes in isorenieratane concentrations were found (Fig. 6). This compound is a biomarker of green sulphur bacteria and acts as an indicator of euxinic conditions in the photic zone of the water column^3,38^. Two maxima in isorenieratane concentrations were identified (Table 2 and Fig. 6). The first is in the lower part of the section (around bed Kow 100), reaching 25 µg/g TOC and the second, smaller maximum (±10 µg/g TOC) in the upper part of the section. Moreover, isorenieratane closely correlates with the 2,3,6−/3,4,5-diaryl isoprenoid, i.e., the so called palaeorenieratane (R^2^ = 0.95), which usually co-occurs with isorenieratane in Palaeozoic sedimentary rocks (e.g.^4,42–49^). Additionally, there is a strong correlation with the concentration of aryl isoprenoids, which are compounds that are degradation products of isorenieratane (e.g.^3,48,50^) (R^2^ = 0.7).

In almost all samples the maleimides (1H-pyrrole-2,5-diones), decomposition products of chlorophylls and bacteriochlorophylls, were identified as the abundant group of compounds from the polar fraction. Between maleimides, those treated as of bacteriochlorophyll origin (with 2-methyl-3-*iso*-butyl- configuration; Me,*i*-Bu) were present in almost all samples. However, the Me,*i*-Bu to Me,Et (2-methyl-3-ethyl-) maleimide ratio^40,51,52^ does not correlate with the isorenieratane and palaeorenieratane concentrations.

### Inorganic geochemical data

The Th/U ratio in almost all carbonate-rich/limestone samples is >1, which is indicative of oxic conditions. Only three samples from the lower part of the investigated section reached Th/U ratio values below 1, which imply dysoxic bottom water conditions. The lower values of the Th/U ratio in shales (<3) are indicative of oxygen depleted conditions in the whole analysed section (Fig. 6). The V/Cr ratios range from 1.21 to 9.03 (Table 3); these values are indicative of oxic through dysoxic to anoxic conditions (e.g.^5,53^). The V/Cr ratio shows a good correlation with Mo as well as the isorenieratane contents. Additionally, the C_org_/P ratio also closely corresponds with these data (Fig. 6 and Table 3). The maximum values of the C_org_/P ratio around bed Kow 101 (first postulated anoxic interval) reached from 116.92 to 212.47, while in the second anoxic interval located between Kow 158 and Kow 162, the ratios reached values from 193.16 to 768.5 C_org_/P, respectively. All results are presented in Table 3 and in Fig. 6.

**Table 3:** Geochemical data of the samples from the lower Famennian Kowala section: TOC, TS, P, U, Th, V, Cr, Mo content and values of the Th/U, V/Cr and Corg/P ratios. Limestones are marked in grey.

Sample	TOC [%]	TS [%]	P (%)	U (ppm)	Th (ppm)	V (ppm)	Cr (ppm)	Mo (ppm)	Th/U	V/Cr	C_org_/P
Kow190A	3.54	0.05	0.06	3.4	6.9	195	61.58	2.2	2.03	3.17	57.96
Kow 186	3.65	0.15	0.13	2.5	7	96	61.58	0.8	2.80	1.56	27.86
Kow 183	2.11	0.03	0.07	1.2	2.2	36	20.53	1.1	1.83	1.75	28.46
Kow 182	3.39	0.02	0.03	2.6	6.2	119	54.74	0.6	2.38	2.17	129.36
Kow 176	1.56	0.06	0.04	2.7	5.5	115	47.89	0.4	2.04	2.40	39.80
Kow 170	4.63	0.03	0.10	2.7	5.7	84	54.74	0.5	2.11	1.53	48.27
Kow 167	0.63	0.12	0.05	1.1	1.9	33	27.37	0.2	1.73	1.21	13.07
Kow 164	1.91	0.38	0.07	3.3	6.4	157	61.58	0.8	1.94	2.55	29.13
Kow 162	3.55	0.01	0.02	3.3	6.1	249	54.74	2.3	1.85	4.55	203.12
Kow 161	3.35	0.05	0.00	1.5	2.2	90	20.53	2.4	1.47	4.38	768.50
Kow 158	3.37	0.02	0.02	3.3	5.7	225	41.05	2.3	1.73	5.48	193.16
Kow 151	2.15	0.01	0.02	1.7	3.7	60	34.21	0.4	2.18	1.75	123.45
Kow 150	3.83	0.03	0.07	3.3	6.2	117	54.74	0.7	1.88	2.14	51.62
Kow 144	3.62	0.04	0.08	2.7	5.8	113	54.74	0.9	2.15	2.06	46.08
Kow 143	1.27	0.01	0.01	1.1	2	46	13.68	0.3	1.82	3.36	97.03
Kow 138	3.18	0.01	0.07	2.9	6.8	121	54.74	0.3	2.34	2.21	42.88
Kow 132	2.06	0.09	0.09	1.6	3.9	62	34.21	0.7	2.44	1.81	23.58
Kow 124	2.93	0.04	0.06	3.2	5.5	108	54.74	0.4	1.72	1.97	47.89
Kow 116	1.20	0.00	0.10	2.6	6	100	54.74	0.4	2.31	1.83	11.97
Kow 115	2.84	0.00	0.02	1.2	1.8	43	20.53	0.5	1.50	2.09	130.20
Kow 112	3.61	0.01	0.02	2.7	4.3	156	47.89	0.7	1.59	3.26	165.57
Kow 108	2.32	0.39	0.03	2.7	5.9	260	61.58	1.4	2.19	4.22	88.41
Kow 107	0.48	0.32	0.01	0.6	1	47	20.53	3.1	1.67	2.29	36.47
Kow 103	0.73	0.09	0.01	0.8	1.3	57	20.53	0.8	1.63	2.78	55.80
Kow 102	4.64	0.03	0.02	2.9	5.7	200	47.89	1.3	1.97	4.18	212.47
Kow 101	2.50	0.08	0.01	1.4	1.1	27	6.84	3.2	0.79	3.95	191.09
Kow 100	3.57	0.39	0.03	3.2	6	156	47.89	2.7	1.88	3.26	116.92
Kow 98	1.62	0.36	0.04	2.8	6.2	142	54.74	1.7	2.21	2.59	41.34
Kow 90	2.31	0.52	0.16	3.7	8.1	90	61.58	0.4	2.19	1.46	14.71
Kow 82	3.65	0.01	0.08	11.5	5.7	162	47.89	0.3	0.50	3.38	43.97
Kow 75	1.76	0.00	0.02	4.2	3.4	247	27.37	2.1	0.81	9.03	100.87
Kow 74	4.36	0.02	0.09	11.2	4.5	115	41.05	0.3	0.40	2.80	47.56
Kow 71	4.47	0.02	0.06	1.8	1.7	52	13.68	0.4	0.94	3.80	78.75
Kow 64	3.02	0.00	0.13	8.5	4.5	142	47.89	0.3	0.53	2.96	23.06

## Discussion

### Changes of sedimentary conditions

Despite the monotonous lithology, similar TOC content through the entire section and only minor fluctuations between the algae and bacteria as a kerogen contributors (see steranes to hopanes ratio in Table 2), organic and inorganic proxies indicate significant changes in depositional conditions and abundance of some fossil groups. There are two visible spikes of isorenieratane, palaeorenieratane and aryl isoprenoid concentrations (Fig. 6; Table 1). The first is located in the lowermost part of the section, around sample Kow 101, and the second is in the upper part, between samples Kow 158 and Kow 162 (Fig. 6). Similar patterns were observed for the Mo concentration and V/Cr ratio, which reached their maxima at the same stratigraphic levels (Fig. 6). The third, smaller isorenieratane spike that correlated with higher concentrations of Mo, V/Cr and C_org_/P ratios is observable in the lowermost part of the section in sample Kow 75 (Fig. 6). Conversely, inorganic proxies based on the uranium (Th/U and U_autig_) and maleimide ratios are not in agreement with the above parameters. The discrepancy between the U proxies and Mo concentration and the V/Cr ratio in the upper part of the section is unclear, especially since all these parameters work closely together in the other Upper Devonian sections from the Kowala quarry, such as those containing the *Annulata* or Hangenberg events^5,42^. Notably, part of the uranium was transported to the basin with a detrital fraction, which, in consequence, changed the ratio between the detrital Th and U connected with primary organic matter. However, the lack of correlation between U and Al (R^2^ = 0.11) does not confirm this mechanism as a reliable factor. The other, more likely explanation of disagreement between Th/U and the other proxies is based on the assumption, that the euxinic zone was located in the water column but did not reach, or only periodically reached, the sea-floor (as it was shown in^43^). Uranium was partially diluted from the sediment after deposition during oxic periods, while Mo connected with pyrite framboids^54^ formed in the water column and survived intermittent oxygenation on the seafloor. Particulate shuttle cannot be excluded as a process driving Mo concentration, but this element does not correspond to the correlation between inorganic and organic indicators.

The lack of correlation between the isorenieratane concentration and the Me,*i*-Bu to Me,Et maleimide ratio is much easier to explain. The origin of Me,*i*-Bu-maleimides is connected with green sulphur bacteria while Me,Et-maleimides are degradation products of both chlorophyll and bacteriochlorophyll^40,51^. This implies that Me,*i*-Bu to Me,Et maleimide ratio values are not only dependent on green sulphur bacteria (GSB) blooms but also on the flowering of algae and other marine microorganisms that generate chlorophyll. Thus, Me,*i*-Bu-maleimides are useful indicators of GSB occurrence, but the Me,*i*-Bu to Me,Et maleimide ratio does not necessarily illustrate the intensity of euxinia.

Gammacerane, an indicator of water column stratification^55^ was found in the all investigated samples and the values of G/H ratio are quite stable across the section (Table 2). This implies, that anaerobic ciliates, which are precursors of gammacerane^55^, were constantly present in water column and most possibly fed on green sulphur bacteria. The presence of gammacerane also indicates a permanent stratification of water column in the aftermath of F/F crisis.

The next proxy that is in accordance with the other proxies used here is the C_org_/P ratio. The values of the C_org_/P ratio, ranging from 30 to 150, are characteristic for high productivity and periodically oxygen-restricted conditions (see^56^), while the higher values of the C_org_/P ratio (>150) are indicative for high-productivity and permanent anoxic conditions on the seafloor. In the case of the investigated section, higher values of C_org_/P are in agreement with the Mo, V/Cr and isorenieratane values, thus confirming a higher productivity and seafloor euxinia (Fig. 6).

Based on isorenieratane, palaeorenieratane and aryl isoprenoid concentrations, as well as the Mo concentration, V/Cr and C_org_/P ratio values, the following scenario of depositional conditions during the *triangularis* zone of the early Famennian can be presented. At the first stage of deposition, conditions were dysoxic to anoxic on the seafloor with photic zone euxinia (PZE) reaching (periodically?) the bottom waters. Then, the conditions became more aerobic, which took place between sedimentation of the layers K112 and K151. The second event saw anoxia/euxinia occurring between the formation of the layers K158 and K167. During sedimentation of the uppermost part of the section, oxygenation of the bottom waters prevailed again, while the water column above may have still witnessed some PZE. The episodes with domination of euxinia in the bottom part of the water column are shaded on Fig. 6.

### Relationship between redox changes and fossil abundance

Depositional conditions are an important factor controlling the state of fossil preservation^57^, and, euxinia/anoxia/dysoxia and water column stratification played an important role in the exceptional preservation of past organisms^2,58^. At Kowala, the lower Famennian section investigated here is regarded as an example of a conservation deposit, possessing well-preserved, abundant and diverse assemblages of arthropods, fish and non‐biomineralised macroalgae^9,13,16^, even with sporadic cases of soft tissue preservation^59^. Interestingly, this fossiliferous interval falls within the lowermost Famennian *triangularis* Zone that marks the immediate aftermath of the Frasnian-Famennian biotic crisis. During this time interval, oxygen-deficient conditions in the Kowala basin occurred as confirmed through gamma-ray spectrometry^60^, as well as geochemical and petrographic studies^43^. Therefore, taking these findings into account, we have expected that the highest number of exceptionally preserved fossils (especially phosphatised arthropods) occur in horizons characterised by elevated anoxia/euxinia, especially since the PZE has been considered as an important factor in preserving fossils in Upper Devonian Lagerstätte deposits such as the Australian Gogo Formation^2^.

However, we found increased amounts of thylacocephalan arthropods at intervals where conditions were rather suboxic and PZE did not reach the seafloor (Figs 4, 5). These intervals of higher abundance of arthropods especially embrace the section from samples K112 to K128, K142 to K150, and K171 to K175 (non-shaded parts of the section in Figs 4 and 5). In the case of other fossils having originally phosphatic shells such as orbiculoid brachiopods, which are the most abundant fossils occurring throughout the studied section (Fig. 4), such tendencies were not observed. It is possible that these organisms could have colonised the sea-bottom only during short oxygenated pulses in the otherwise suboxic bottom waters (e.g.^5,61,62^,). However, considering their large abundance throughout the studied section, irrespective of the anoxic events, it is even more probable that the orbiculoids were epiplanktonic^63^; these orbiculoids would have drifted in the surface waters attached to algae occurring in the same deposits^16^, or would have functioned as opportunistic bottom dwellers able to thrive in stressed, oxygen-deficient conditions as other linguloid brachiopods known from the Devonian^64^.

Our data show that arthropods (and especially dominant thylacocephalans) were common only during oxic/suboxic periods (Figs 5, 6) with a thin PZE located in the water column above^43^, while more restricted conditions were unfavourable for this group of organisms.

The mode of life of thylacocephalans is still problematic. They have been interpreted either as nektonic predators^65^, benthic ambush predators, or benthic scavengers^66–68^. For some species (mostly protozoeids), nektonic mode of life was proposed on the basis of such features as their small size, elongated carapace and hyperthrophied eyes^69,70^. A nektobenthic mode of life of thylacocephalans instead, has been proposed by various authors (e.g.^67,71^) based on such features as reduced posterior trunk appendages, lack of a flexible abdomen (see^11,70,72^) and their rather thin, poorly mineralised cuticles^68,73^. However the last feature is shared with some nektonic arthropods, like amphipods^74^, so except the last one, these features can be observed in modern nektobenthic crustaceans (both swimming and seafloor-dwelling^75^). Although the anatomy of both raptorial and posterior appendages of the Kowala thylacocephalans remains unknown, the anatomy and architecture of their carapaces were described by Broda and Zatoń^10^. The authors pointed out an additional important feature: the presence of “sensory belts” in the upper and lower margins of the carapace. The authors assumed that these zones, consisting of many organule canals, are the remnants of a highly developed sensory system that can monitor the surrounding space. Such a developed set of sensors could allow these predators to easily find their prey either in the surrounding water column, or hiding on the sea bottom. Such sensors could have also served as anti-predatory “alarm device”. However, taking all these known features into account, we are still not sure about the exact thylacocephalan mode of life. Depending on species, it would probably be either nektonic or necto-benthic organism.

The lack of arthropod fossils in euxinic intervals (Figs 3 and 5), however doesn’t have to be necessarily connected with unfavourable living conditions. Based on modern examples from the Santa Monica Basin, the Black Sea, the Baltic Sea and other basins, anoxic conditions at the seafloor promote phosphorous flux out of the sediment^76–78^. This is in accordance with P concentrations throughout the section, showing its lowermost values exactly within the euxinic levels (Fig. 6). At the same levels, C_org_/P ratio values are also the highest. As shown by Zatoń *et al*.^9^, exoskeletal remains of all arthropods from the Late Devonian of the Kowala quarry are phosphatic. Apparently, a lack of sufficient phosphorous in sediments during the euxinic periods precluded arthropod preservation and in consequence controlled the overall fossilisation processes of the arthropod exoskeletons. The abundance of arthropod exoskeletons in rocks characterised by suboxic conditions during their sedimentation coincides with much higher values of P (Figs 4 and 5). Phosphatisation requires a special microenvironment characterised by a specific pH, redox conditions and a sufficient concentration of P^79–82^. Thus, all parameters promoting phosphatisation of the arthropod cuticle appear to have been present in the Kowala suboxic environment.

The alternative hypothesis, which assumes mass mortality events that may have occurred during euxinic events (e.g.^8^), is not supported in our case. This is primarily because the PZE intervals in the studied section are depleted in fossil arthropods. In conclusion, the post-Frasnian-Famennian crisis interval in the Kowala quarry is rich in the opportunistic benthic linguloid brachiopod *Orbiculoidea*, orthoconic nautiloids and phosphatised remains of arthropods, among which Thylacocephala dominate. As linguloid brachiopods occur throughout the studied section, the arthropods appear to be absent in intervals when PZE was detected. The simultaneous drop in phosphorous content in the euxinic intervals indicates that the absence of arthropods resulted rather from their non-preservation due to low P content than from changes in palaeoenvironmental conditions. Thus, in this case, euxinic/anoxic conditions negatively influenced the preservation of arthropod exoskeleton via phosphatisation instead of promoting their fossilisation as has been suggested in an earlier study^2^. Apparently, PZE conditions were not universal for fossil preservation through phosphatisation. Thus, any interpretations concerning the abundance of fossils within euxinic horizons should be treated with caution, and taphonomic causes may be crucial, primary factors in controlling the presence or absence of fossils in the rock record.
